# Carcinome à cellule vitreuse du col de l'utérus: à propos d'un cas et revue de littérature

**DOI:** 10.11604/pamj.2015.22.55.7607

**Published:** 2015-09-18

**Authors:** Ihssane Hakimi, Abdelghani Zazi, Hafsa Chahdi, Khalid Guelzim, Jaouad Kouach, Myabdellah Babahabib, Myehdi Elhassani, Driss Moussaoui Rahali, Mohammed Dehayni

**Affiliations:** 1 Service de Gynécologie Obstétrique, Hôpital Militaire d'Instruction Mohammed V, Rabat, Maroc; 2 Service d'Anatomie Pathologique, Hôpital Militaire d'Instruction Mohammed V Rabat, Maroc

**Keywords:** Carcinome à cellule vitreuse du col utérin, Meta analyse, col de l′utérus, Glassy cell carcinoma of the cervix, Meta analysis, cervix

## Abstract

Le carcinome à cellule vitreuse du col de l'utérus est un type de histologique rare de cancer du col de l'utérus qui survient à un âge plus jeune, et s'associe au risque élevé d’échec thérapeutique et le pronostic est plus mauvais en comparaison au type cellulaire squameux. La radiothérapie est associée au risque diminué de récidive. Le but de cette étude est de récapituler à travers d'une observation et une revue de littérature les données sur l'incidence, le comportement clinique et la survie globale de patients avec le carcinome à cellule vitreuse du col de l'utérus.

## Introduction

Le cancer du col de l'utérus est le 14^ème^ cancer fréquent dans USWOMEN dont 80% de cas arrivant des pays en voie de développement. Pour tous les cancers du col de l'utérus, indépendants de type de histologique, la survie en général de 5 ans s'approche de 75%. Des carcinomes cellulaires squameux représentent 90% avec la majorité de 10% restants étant des adénocarcinomes. Ces dernières années, l'incidence d'adénocarcinomes a augmenté [1, 2]. Le carcinome à cellule vitreuse du col de l'utérus a été reconnu comme un sous-type de cancers du col de l'utérus adénosquameux mixtes. D'autres cancers du col de l'utérus adénosquameux mixtes incluent adénocarcinome mûr non différenciée. Des caractéristiques des cellules vitreuses sont aussi été identifiées comme de larges cellules non différencié non kératinisés des cancers du col de l'utérus [3]. Le carcinome à cellule vitreuse du col de l'utérus est connu historiquement de répondre mal à la radiation et la chirurgie avec les taux de survie les plus faibles [4]. Des directives actuelles pour le traitement de cancers du col de l'utérus, y compris à cellule vitreuses sont les même que de carcinome cellulaire squameux du col de l'utérus. En raison de la rareté de cette maladie et manque d’études prospective, la gestion du carcinome à cellule vitreuse du col de l'utérus n'a pas été spécifiquement définie. Des protocoles de traitement utilisés dans le passé ont des résultats pauvres. Le but de ce méta analyses est de rapporter l'incidence, des signes cliniques et la survie globale de cette variante de tumeur rare.

## Patient et observation

**Figure 1:**
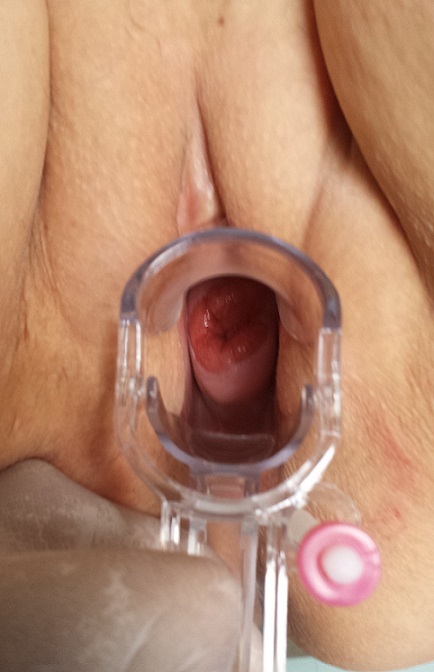
Le processus tumoral cervical

**Figure 2:**
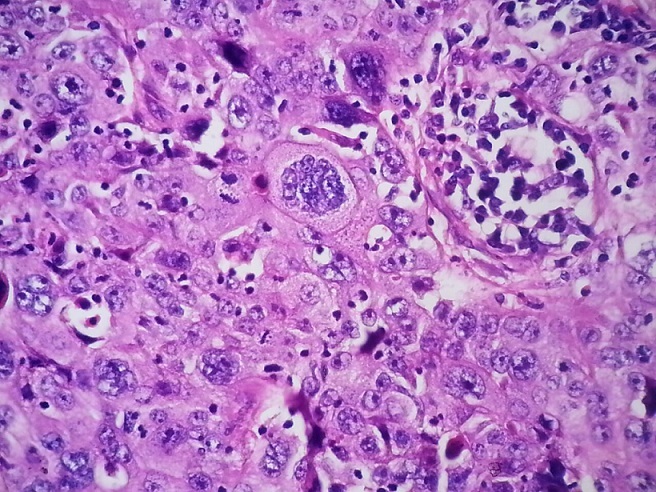
Coloration émétine eodine de grossissement x40 montrant une prolifération tumorale faites de nappe de cellules atypiques

Mme A.A âgée de 41 ans, grande multipare, sans antécédents pathologiques notables, admise dans un tableau de métrorragie poste coïtale, à l'examen on trouve un col ulcéré de 4 cm (Figure 1) avec atteinte des paramètres proximaux des deux cotés, cul de sac vaginaux intacts et cloison recto vaginale libre. Une IRM pelvienne confirme l'examen clinique avec un stade IIB proximal. La patiente a bénéficié d'une biopsie qui revient en faveur d'une tumeur à cellule vitreuse (Figure 2) pour laquelle elle a reçu 25 séances de radiothérapies et la curiethérapie et six cures de chimiothérapie à base de cisplatin. Une IRM de contrôle qui montre une régression totale du processus tumorale avec absence d'anomalie de signale intra utérin et sans adénopathies profondes ni épanchement. La patiente a ensuite subi une colpo hystérectomie élargie avec lymphadénectomie. La surveillance à un an par des examens cliniques et de frottis de contrôle ne montre pas de signe de récidives.

## Discussion

Glucksmannn et al est le premier à décrire des carcinomes à cellules vitreuses en 1956 comme ayant trois caractéristiques distinctes: 1. cytoplasme d'apparence de verre dépoli, 2. Parois cellulaires distinctes et 3. Grands noyaux avec nucléole proéminent [3, 4]. La classification pathologique de cette tumeur est stvariable et histologiquement présente des variabilités dans des résultats rapportés. Nunez et al, Pak a exposé le carcinome à cellule vitreuse du col de l'utérus comme de grande cellule, non-kératinisées [5–7]. Tamimi et al a discuté la possibilité de d’étendre la classification de cellule vitreuse comme étant une caractéristique trouvée dans des carcinomes non différenciés et des grands cancers cellulaires non-kératinisées [8]. Costa et al. différencie le carcinome à cellule vitreuse du col de l'utérus des cancers adenésquameus avec la prédominance des caractéristiques des cellulaires vitreuses (N85%). En général, il semble y avoir un spectre de caractérisation pathologique pour le carcinome à cellule vitreuse du col de l'utérus qui devrait être considéré comme une forme mal différenciée de carcinome adénosquameux mixte. Il est probable que la mesure de participation cellulaire vitreuse dicte la réponse au traitement incluant la radiothérapie. Dans cette meta-analyse, les patients avec le carcinome à cellule vitreuse du col de l'utérus sont plus jeunes (l’âge moyen 45) en comparaison de tous les cancers du col de l'utérus dans notre observation l’âge de notre patiente est 41 ans. La majorité de patients était blanche (64.9%) et il n'y avait aucun impact significatif sur la survie globale. Les patients diagnostiqués étaient principalement noir, alors que dans notre observation la patiente est blanche. La présentation clinique du carcinome à cellule vitreuse du col de l'utérus est semblable au cancer du col de l'utérus envahissant avec le saignement vaginal qui semble être le symptôme prédominant. Le comportement clinique incluant la réponse au traitement de ces carcinomes est difficile de caractériser avec des informations limitées rapportés par la série. La distribution d’étape semble être semblable à celui de carcinome cervical squameux et la majorité des patients (79%) a été diagnostiquée au stade I ou II (36) dans notre observation la patiente était de stade IIBa. La survie globale a été résolument diminuée et La survie médiane pour stade II était 25 mois et a diminué à 3 mois pour le stade IV de la maladie. Tandis que le résultat global du carcinome à cellule vitreuse du col de l'utérus est pauvre, la maladie de stade précoce, particulièrement quand elle est localisée au col de l'utérus, peut avoir des taux de guerrison comparables à celui de tous cancers du col de l'utérus.

Gris et al. a rapporté une survie globale de 86% pour stade I, le taux de survie global était 22% [9]. En raison du manque de preuve des recommandations de traitement, les patients avec carcinome à cellule vitreuse du col de l'utérus ont reçu des traitements divers incluant la chirurgie, la radiation ou la chimiothérapie aux combinaisons diverses [10–13]. La radiosensibilité est soulignée dans l’étude par Randall et al. la majorité des stades traitées par chirurgie (44.1%) ou chirurgie suivie par radiothérapie (32.2%). À 11.5% des patients, tous les trois modalités thérapeutiques incluent la chirurgie, la radiothérapie et chimiothérapie sont utilisées. La majorité des stades II et III des patientes sont traitées par radiothérapie seulement (46.9% et 53.8%, respectivement). Le taux de récidives par ce traitement était le plus haut parmi des patients traités seulement avec la chirurgie (32.7%), en comparaison des patients a traité avec la chirurgie suivie par la radiothérapie (11%). L'utilisation de chimiothérapie pour optimisation des résultats à la radiothérapie dans le cancer du col de l'utérus est soutenue par sept grandes études randomisées qui ont démontré une réduction marquée du taux de récidives et l'amélioration significative de la survie quand la chimiothérapie a été utilisée simultanément avec la radiothérapie, utilisant la chimiothérapie par cisplatin [14, 15]. Les patients avec le carcinome cellulaire squameux, adénocarcinome et des variantes histologiques rares ont été inclus dans ces études. De façon intéressante, dans le stade II de la maladie la combinaison de radiation et la chimiothérapie semble améliorer le résultat de survie. Le taux de récidives tardives était 33% pour des patients a traité avec la chirurgie, 3% pour des patients ont traité avec la radiation et 0% pour des patients ont traité avec radio-chimiothérapie [16, 17]. Dans l’étude par Duenas et al. la chimiothérapie adjuvante avec cisplatin et gemcitabine a diminué les récidives et a amélioré en général la progression la survie globale des malades avec stade II-IV le cancer du col de l'utérus aux dépens de la toxicité accrue mais cliniquement gérable [16]. Des patients qui ont été inscrits dans cette étude, 6% ont été diagnostiqués avec les types divers d'adenocarcinoma incluant le carcinome à cellule vitreuse du col de l'utérus. Littman.et al a rapporté que la résistance au traitement peut être due au développement rapide de la tumeur, et la fréquence des métastases éloignées et la diminution de la radiosensibilité [18]. Tsukahara et al a attribué bas radiosensibilité à une augmentation relative de cellules de tumeur anoxiques, des parois cellulaires épaisses et l'attache forte par des fibres de collagène observées dans l'etude histologic des carcinomes à cellule vitreuse du col de l'utérus des échantillons [19]. Depuis la publication par Littman et les groupes de Tskukahara, plusieurs études se sont concentrées sur radiosensibilité d'adénocarcinomes et des cancers du col de l'utérus adénosquameux. Les études ont montré que les adénocarcinomes et des cancers adénosquameux ont plus de radiorésistances comparées avec les cancers cellulaires squameux du col de l'utérus [20]. La différence dans la survie entre les types d'histologiques de cancers du col de l'utérus ne montrant aucune différence significative dans la survie par le type d'histologique. La plus grande étude avec n = 11,157 par Shingletonet et al n'a pas trouvé de différences significatives de la survie de 5 ans entre le carcinome cellulaire squameux, adénocarcinome, ou le carcinome adénosquameux à part le stade II des patients [20]. HPV à haut risque a été détecté dans la majorité d’échantillons de carcinome à cellule vitreuse du col de l'utérus GCCC analysés; sans aucune conclusion sur l'association ou la signification pronostique. Les découvertes sur l’‘strogène et l'expression de récepteur de progestérone ont été variables [19, 20].

## Conclusion

Pour conclure, le carcinome à cellule vitreuse du col de l'utérus est une variante de tumeur rare avec la présentation de patients à un âge generalement plus jeune que la population générale avec le cancer du col de l'utérus. La distribution de stades est semblable à celui de carcinome cellulaire squameux du col de l'utérus. Survie de Cinq année est inférieure en comparaison de tous les cancers du col de l'utérus pour le stade II et IV. Alors que pour le stade III, le taux de survie globale est comparable. La survie En général de 5 ans pour toutes les stades est beaucoup plus basse comparé à tous les cancers du col de l'utérus (54.8% contre 75%). La radiothérapie comme le traitement adjuvant principal ou postopératoire est associée au contrôle local amélioré. La chimiothérapie devenant sensible pourrait améliorer les résultats. Étant donné le risque élevé de récidives, on devrait donner la considération à la chimiothérapie adjuvante.
